# Exploring Neighborhoods in the Metagenome Universe

**DOI:** 10.3390/ijms150712364

**Published:** 2014-07-14

**Authors:** Kathrin P. Aßhauer, Heiner Klingenberg, Thomas Lingner, Peter Meinicke

**Affiliations:** Department of Bioinformatics, Institute for Microbiology and Genetics, University of Göttingen, 37077 Göttingen, Germany; E-Mails: kathrin@gobics.de (K.P.A.); heiner@gobics.de (H.K.); thomas@gobics.de (T.L.)

**Keywords:** metagenomics, functional profile, taxonomic profile, metagenome comparison

## Abstract

The variety of metagenomes in current databases provides a rapidly growing source of information for comparative studies. However, the quantity and quality of supplementary metadata is still lagging behind. It is therefore important to be able to identify related metagenomes by means of the available sequence data alone. We have studied efficient sequence-based methods for large-scale identification of similar metagenomes within a database retrieval context. In a broad comparison of different profiling methods we found that vector-based distance measures are well-suitable for the detection of metagenomic neighbors. Our evaluation on more than 1700 publicly available metagenomes indicates that for a query metagenome from a particular habitat on average nine out of ten nearest neighbors represent the same habitat category independent of the utilized profiling method or distance measure. While for well-defined labels a neighborhood accuracy of 100% can be achieved, in general the neighbor detection is severely affected by a natural overlap of manually annotated categories. In addition, we present results of a novel visualization method that is able to reflect the similarity of metagenomes in a 2D scatter plot. The visualization method shows a similarly high accuracy in the reduced space as compared with the high-dimensional profile space. Our study suggests that for inspection of metagenome neighborhoods the profiling methods and distance measures can be chosen to provide a convenient interpretation of results in terms of the underlying features. Furthermore, supplementary metadata of metagenome samples in the future needs to comply with readily available ontologies for fine-grained and standardized annotation. To make profile-based *k*-nearest-neighbor search and the 2D-visualization of the metagenome universe available to the research community, we included the proposed methods in our CoMet-Universe server for comparative metagenome analysis.

## 1. Introduction

With the rapidly increasing number of sequenced metagenomes in current databases it has become important to be able to compare novel metagenomic data with the existing data on a large scale [1,2]. In particular, the identification of closely related metagenome datasets (“neighbors”) to a newly obtained dataset is of growing importance for downstream analysis. Firstly, inspection of the neighbors and their associated annotations can be used as a final quality control of the dataset and may reveal unexpected flaws of the sampling, sequencing or data processing procedures. For instance, neighbors with unexpected habitat labels may indicate some contamination of the sample [3]. Secondly, related metagenome datasets in the neighborhood can be used as additional data sources for comparative analyses. Similar to biological replicates in gene expression analysis or homology extension in sequence analysis, the neighbors may be used for a statistical characterization of variations. However, manually identifying neighboring datasets on the basis of metadata can be misleading with the currently available coarse-grained and non-standardized annotation categories. If, for instance, the existing habitat annotations are used for sample selection, it is unclear which metagenomes are good neighbors for a data-driven comparative analysis, in particular, if a habitat label is rather abundant or rather sparse within the database. 

Because metagenomic data usually consists of huge collections of short anonymous sequences, the comparison of two metagenomes is notoriously difficult. In analogy to comparative genomics a comparison may be conducted on a sequence-by-sequence basis to identify all pairwise similarities between two metagenomic data sets [4]. However, the computational cost for all pairwise sequence comparisons between a new query data set and n metagenomes in a database is prohibitively expensive due to the average size of a single file that may comprise several millions of sequences. Therefore, instead of the sequences it is reasonable to compare feature profiles that can represent relevant aspects of the functional and taxonomic composition of metagenomic sequence data [5,6,7,8,9,10,11]. But so far, it is unclear what kind of features and which metrics are most suitable for the comparison of metagenomes. 

We present here a study of profile-based methods for nearest neighbor identification according to metagenome habitat annotation, using a broad spectrum of profile representations and distance metrics. Our results indicate that taxonomic as well as functional profiles can be used to retrieve related metagenomes in a database with a high confidence. Furthermore, we found that several standard metrics such as the City block or Euclidean distance are well-suitable for the identification of biologically meaningful nearest neighbors. In this context, we also investigated the performance of dimensionality reduction methods for visualization of the “metagenome universe”, where unsupervised kernel regression [12] showed the best representation in terms of neighborhood conservation. 

## 2. Results and Discussion

The rapidly growing number of publicly available metagenomes nowadays requires efficient tools to compare and relate a novel metagenome to those in databases. In this study, we investigate the possibility to detect and visually explore metagenomic neighbors based on taxonomic, functional and metabolic profiles. In the following, we will first present the results of our evaluation of neighborhood accuracy and then discuss the opportunities and difficulties of a dimensionality-reduced representation of metagenome profiles for visual inspection. 

### 2.1. Neighborhood Accuracy

The neighborhood accuracy measures the fraction of metagenomes with the same habitat label among the *k* nearest neighbors as obtained from a leave-one-out cross-validation. It is an estimator of the posterior probability to find related metagenomes within a local neighborhood of the profile space. For profile-based approaches the achievable accuracy depends on the particular feature space and the distance metrics that is used for comparison. 

#### 2.1.1. HMP Collection

The Human Microbiome Project (HMP [13], see also Section 3.1.1.) provides high-quality sequencing data and a consistent habitat annotation of metagenomes in terms of distinct body sites. Therefore, we expect only a small overlap of HMP samples from different body sites, indicating a suitable benchmark dataset for the evaluation of metagenome profiling methods. Originally, the phylogenetic, functional, and metabolic profile of the HMP data have been investigated by means of the HMP Unified Metabolic Analysis Network (HUMAnN) pipeline [15], the Metagenomic Phylogenetic Analysis (MetaPhlAn) tool [14] and a Gene Ontology (GO) Slim analysis. Besides these annotations we also used different taxonomical, functional and metabolic profiling methods as described in Section 3.2.1. and evaluated the *k* nearest neighbors according to Section 3.3. 

**Figure 1:**
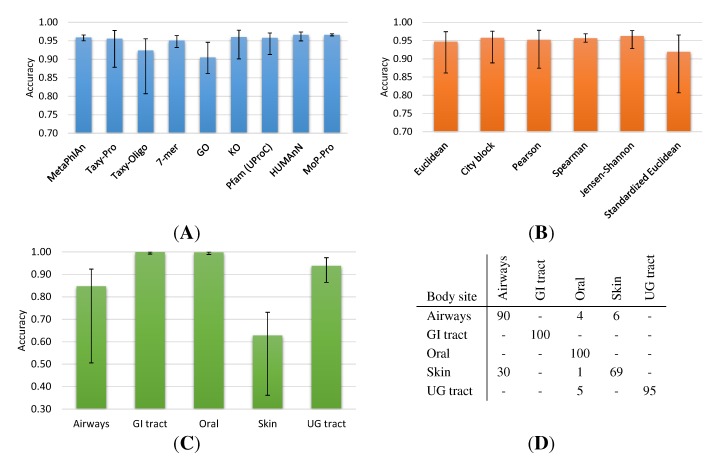
Neighborhood accuracy on Human Microbiome Project (HMP) data for different profiling methods, metrics and body sites. (**A**) Accuracy of profiling methods with average/minimum/maximum over six different metrics; (**B**) Accuracy of distance metrics with average/minimum/maximum over all nine profiling methods; (**C**) Body site-specific accuracy for City block metric averaged over nine profiling methods; (**D**) Confusion matrix of neighborhood evaluation for different body sites according to UProC protein domain profiles and City block metric. Values represent rounded percentages and entries lower than 0.5 are omitted.

Figure 1 shows the neighborhood accuracy on the HMP dataset for different profiling methods, metrics and body sites. Figure 1A indicates that in general a high fraction (≈90% to 97% on average) of equally-labelled neighbors can be detected by all methods. Here, the MetaPhlAn and MoP-Pro methods show very little variation of the accuracy with respect to the underlying profile distance measure. On the other side, Taxy-Oligo and GO show a relatively low accuracy on average and are much more susceptible with respect to the distance metric. The GO Slim profile space has the lowest dimensionality and it seems to require a nonlinear metric or a more suitable normalization, while the relatively low accuracy of Taxy-Oligo is mainly caused by the standardized Euclidean metric (see Figure S1) that seems to be unsuitable for the corresponding profiles. This distance measure showed the lowest average accuracy for most of the methods (see Figure 1B), but as an exceptional case it did improve the performance of the 7-mer approach (see Figure S1). 

Figure 1B also indicates the Spearman metric as the most robust distance measure with respect to the choice of the profiling method, however, the conversion of category counts to ranks for the calculation of this metric is problematic when only a few counts are present for many categories. Except for the GO profile space, the City block metric generally showed a high accuracy and allows a fast calculation of distances as well as an intuitive interpretation. Further inspecting the City block results, we found that three HMP body sites (“GI tract”, “UG tract”, “Oral”) allow a high neighborhood accuracy for all methods, while the “Skin” and “Airways” categories show a low average accuracy and a large variation with respect to the utilized method (Figure 1C and Figure S2). The low accuracy cannot be attributed to particular profiling methods or metrics (see Figures S3 and S4) and thus indicates a systematic overlap of categories. Indeed, the “Skin” body site comprises only a few datasets (26 samples) and the confusion matrix of the neighborhood evaluation (Figure 1D) indicates a large fraction of neighbor misassignments to the “Airways” category. Because the Airways samples have been taken from nose regions there might be a natural overlap with skin-associated microbial communities. 

Further characterization of the overlap in terms of the profile space distances turned out to be difficult because the corresponding neighborhood patterns can vary considerably. To illustrate this variation for the Airways and Skin body sites, we represented the metagenome neighborhood of two query metagenomes from the Airways category in terms of multidimensional scaling (MDS) plots and a hierarchical clustering analysis (HCA) of the neighboring functional profiles (see Figure S5). Based on the evaluation of *k* = 10 neighbors in the UProC domain profile space using the City block metric, the two Airways query metagenomes are in one case assigned to the right habitat (6 correct labels) and in the other case misclassified (4 correct labels). In the first case (Figure S5A,B) the five nearest neighbors of the query are grouped into a cluster consisting of four correctly (Airways) and one incorrectly (Skin) assigned neighbor(s). Another cluster shows a mixed composition of metagenomes from the Skin and Airways categories. In the second example (Figure S5C,D) the Airways query metagenome is grouped within a cluster of four Skin samples. However, another cluster consisting of four Airways samples and one Skin sample is located nearby. Although these examples indicate the difficulties of overlapping habitats, they do not allow inferences about the reasons of possible misclassifications. Here, further statistical analysis based on taxonomic, functional or metabolic features of the metagenomic neighbors would be necessary. 

#### 2.1.2. Metagenome Universe Collection

To investigate whether the findings on the HMP dataset collection could be reproduced with a more diverse range of biomes, we analyzed a set of 1745 publicly available metagenomes associated with twelve different habitat categories (“metagenome universe”, see Section 3.1.2. for details). Here, we expect that the overlap of categories is larger than in the HMP collection, since not all labels actually describe distinct environments. We excluded MetaPhlAn and the HUMAnN pipeline from the analysis for computational reasons and the Taxy-Oligo method because of its shortcomings regarding the profiling of viral metagenomes [9]. 

**Figure 2:**
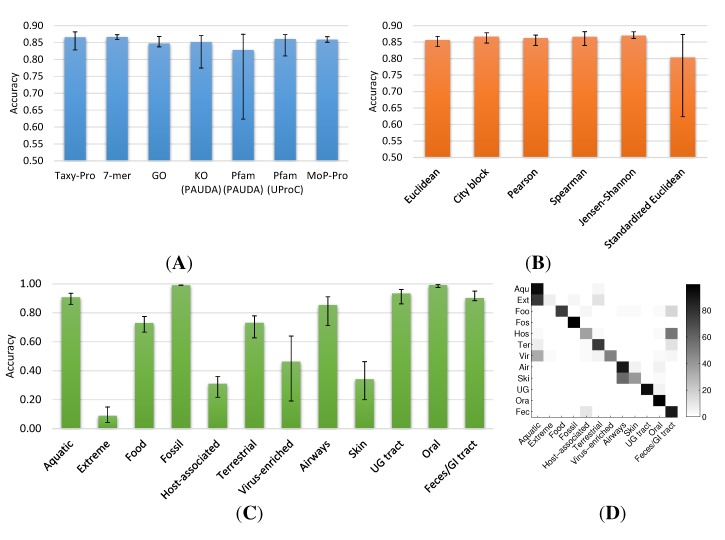
Neighborhood accuracy on metagenome universe collection for different methods and habitats. (**A**) Accuracy of profiling methods with average/minimum/maximum over six different metrics; (**B**) Accuracy of distance metrics with average/minimum/maximum over all seven profiling methods; (**C**) Habitat-specific accuracy for City block metric averaged over seven profiling methods; (**D**) Heatmap of confusion matrix for different habitats according to UProC protein domain profiles and City block metric. Habitat labels on *y*-axis abbreviated to three letters.

Figure 2 shows the neighbor detection performance on the metagenome universe collection for different profiling methods, metrics and habitats. In general, the average neighborhood accuracy of all methods is slightly lower (∼83% to 87%) than on the HMP dataset (see Figure 2A). In particular, the protein alignment using a DNA aligner (PAUDA) method for detection of significant Pfam protein domains and Kyoto Encyclopedia of Genes and Genomes (KEGG) orthologs shows a substantially lower accuracy and higher variation. This is mainly caused by use of the standardized Euclidean metric (see Figure 2B), which seems to be susceptible to small Pfam/KO counts resulting from the low sensitivity of the PAUDA similarity detection. 

Concentrating on more robust metrics such as the City block and Spearman distance, we observe large differences in the ability to correctly detect neighbors for different habitat categories (see Figure 2C, Figures S6 and S7). In particular, the categories “Extreme”, “Virus-enriched”, “Host-associated” and “Skin” indicate low accuracies and/or large variations with respect to the utilized profiling method. While the performance of the oligonucleotide-based 7-mer method noticeably decreases for virus-enriched and skin metagenomes, the GO method shows particularly low accuracy for the host-associated category. 

Considering the habitat annotation of the metagenomes, the difficulties of the evaluation of neighborhood detection become apparent. For instance, the “Extreme”, “Virus-enriched” and “Host-associated” categories just provide a rather unspecific labelling of datasets. A closer look at the confusion matrix associated with our neighborhood evaluation using UProC indicates a systematic overlap of the “Extreme” and “Virus-enriched” categories with the “Aquatic” habitat and of “Host-associated” environments with the “Feces/GI tract” category (see Figure 2D and Table S1). 

This can be well explained by the natural overlap of the annotation, which does not define mutually exclusive habitat categories in this case. 

### 2.2. Visual Exploration of the Metagenome Universe

**Figure 3:**
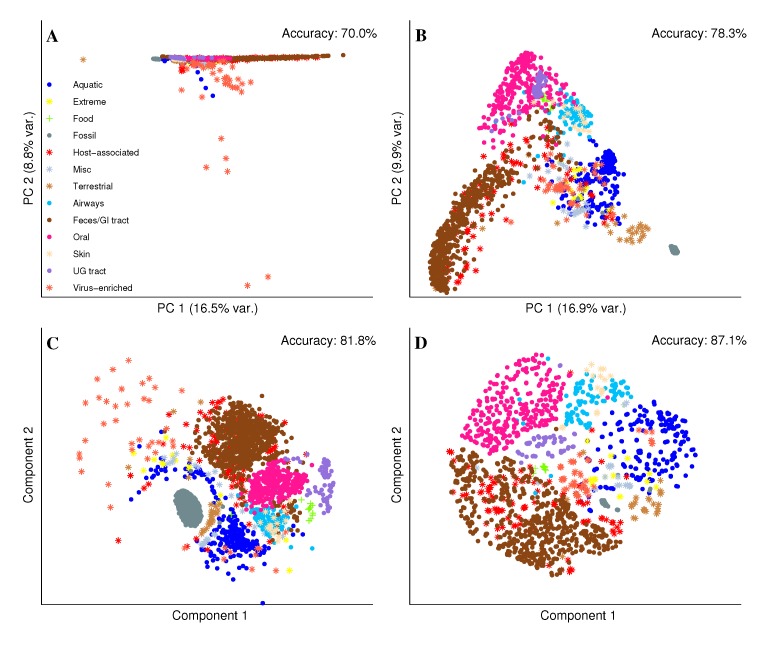
2D representation of metagenome universe for different dimension reduction methods using UProC protein domain profile space. Markers represent metagenome datasets with colors corresponding to habitat labels as provided in legend in subfigure (**A**) Principal component analysis (PCA) using Euclidean metric with dimension-specific variance in parantheses; (**B**) Multidimensional scaling (MDS) using City block metric with dimension-specific variance in parantheses; (**C**) Sammon mapping using City block metric; (**D**) Unsupervised kernel regression (UKR) using City block metric.

The objective of the dimensionality reduction was to obtain a two-dimensional representation of the comprehensive metagenome collection (“metagenome universe”) for scatter plot visualization. In a suitable scatter plot, data points appear closer to each other on the plot when they reflect similar properties. Therefore, the adjacent data points should correspond to related metagenomes with the 2D neighborhoods reflecting the habitat labeling. To obtain the scatter plots, we applied different dimension reduction methods to the UProC protein domain profiles of metagenomes. First, we applied classical principal component analysis (PCA) which showed the well-known susceptibility to outliers ([25], see Figure 3). In this case a few virus-enriched metagenomes are spanning the whole scatter plot and one has to zoom into the main part of the distribution to see meaningful neigborhoods. This is also reflected by the 2D Euclidean neighborhood accuracy which is only 72%. Plotting subsequent principal components (e.g., PC 2 and PC 3) against each other or removing a few apparent (viral) outliers did not enhance the overview given by the PCA plot (data not shown). Only the complete removal of viral metagenomes from the database yields a visualization of the metagenome universe with distinguishable clusters according to habitats (see Figure S8). Using a City block distance matrix, classical multidimensional scaling (MDS) shows a more suitable sketch of the distribution with a considerably reduced influence of the virus-enriched metagenomes. This also resulted in an increased neighborhood accuracy of 78.3% for the MDS coordinates which show an interesting distribution. The shape corresponds to the so-called horseshoe effect which is well-known for MDS and occurs when only the distances between nearby points are representative [26]. Thus, we speculate that for unrelated habitats the distance between protein domain profile vectors does not reflect biologically meaningful differences. We also used the City block distances as an input for the Sammon mapping which also shows a good clustering of metagenomes according to their habitat and a slightly increased neighborhood accuracy of 81.8%. The most convincing result we achieved with unsupervised kernel regression which showed the best utilization of the image area and the highest neigborhood accuracy. In this case, the 87.1% accuracy in 2D was nearly as good as for the original space of the high-dimensional Pfam profiles (87.4%). 

## 3. Materials and Methods

In the following, we will describe the datasets and the experimental setup used in this study. First, we will give an overview of the two different collections of metagenome datasets and the methods used to compute the taxonomic, functional and metabolic profiles. Finally, we present the profile distance measures for neighbor detection and the dimensionality reduction methods used for visualization. 

### 3.1. Metagenome Dataset Collections

#### 3.1.1. HMP Collection

The Human Microbiome Project (HMP, [13]) provides an extensive collection of samples from human body sites of healthy individuals for large-scale comparative studies. More than a thousand HMP data sets have been recorded and are publicly available in HMP’s Data Acquisition and Coordination Center (DACC) Project Catalog [29]. From the HMP-DACC website we obtained the available metadata for the metagenomic samples including taxonomic and functional annotations [30]. The taxonomic annotation comprises the results of the Metagenomic Phylogenetic Analysis (MetaPhlAn) tool [14,31]. Further, we used the summary matrix of the Gene Ontology (GO) Slim analysis [32] (“GO Slim Summary File”) and the functional and metabolic reconstruction data as precomputed through the HMP Unified Metabolic Analysis Network (HUMAnN) pipeline [15,33] (“KEGG pathway abundance values—Summary file” and “Enzyme Abundance Data”). 

For our evaluation, we used 750 clinical study-related samples of HMP data as described in [9] (see also Supplementary Information). Furthermore, we restricted our evaluation to those body sites for which at least ten samples were available. The final dataset includes 640 data samples from five major body sites (see Table S2A). 

#### 3.1.2. Metagenome Universe

In addition to the HMP datasets, we used a large collection of publicly available metagenome datasets from the MG-RAST [16] and European Bioinformatics Institute (EBI) online resources [17] to compile a “metagenome universe”. For this purpose, all publicly accessible dataset files from the MG-RAST website [34] were downloaded in December 2012. We selected the FASTA files that passed the MG-RAST quality control filters and removed datasets with less than 1000 hits to Pfam protein domains. We used the metadata annotation of MG-RAST to assign each of the resulting 664 metagenomes to one of twelve habitat categories (see Table S2B). Furthermore, we downloaded the “project.csv” file from the EBI metagenomic projects website [35] and used it to obtain all associated FASTA files that also passed the EBI quality control. After filtering datasets with less than 1000 hits to Pfam domains, 821 of the 1307 samples were used for our reference database. 

To reduce redundancy in the database, we computed Pfam domain profiles of all metagenomes and selected one representative file from datasets with a high profile correlation (>0.995). Furthermore, we assessed taxonomic coverage quality values in terms of the “fraction of domains unexplained” (FDU, see [9]) for all metagenomes and removed those with an FDU value above 0.6. Finally, we removed datasets associated with profiles that had hits to less than 400 different Pfam families from our database. 

As an exception, we did not apply this procedure to virus-enriched metagenomes, *i.e.*, datasets with a high fraction of viral DNA (>20% as measured by Taxy-Pro). The total number of datasets according to habitat categories can be found in Table S2B. A CSV-formatted list containing the metagenome identifiers and habitat labels as used in our evaluation can be found in Supplementary Dataset. 

### 3.2. Profiling Methods

We used a variety of different profiling methods with largely varying dimensionality ranging from 61 (GO) to 16,384 (7-mer oligonucleotide frequencies). The theoretical dimensionality of the different profile spaces and the actual number of non-zero dimensions can be found in Table S3. 

#### 3.2.1. Pfam Protein Domain Annotation

The ultrafast protein classification (UProC) that is part of the CoMet web server [8] was used for computation of the functional profiles according to the Pfam 27 database. The Pfam profiles also served for estimation of taxonomic and metabolic abundances with the protein-based mixture models (Taxy-Pro, MoP-Pro). For metagenome universe datasets we used the Pfam profiles to calculate GO functional profiles according to the HMP GO Slim ontology scheme. For this purpose, we downloaded the Pfam to GO mapping from the GO website [36] and counted all associations of GO Slim terms with Pfam domains detected in a metagenome. 

#### 3.2.2. Taxonomic Profiling

The mixture model-based Taxy approach provides a computationally efficient and direct estimation of taxonomic abundances in metagenomes. Taxy-Oligo [18] and Taxy-Pro [9] apply a mixture model to approximate the overall metagenome distribution of oligonucleotides and protein domain hits, respectively. For the evaluation on HMP data, all reference profiles were obtained from 1912 bacterial and 133 archaeal genomes available in the KEGG database (release 64.0). These genomes were also used for precomputing the organism-specific pathway abundances for the metabolic profiling of metagenomes. For each reference genome we computed oligonucleotide (7-mers) and protein domain signatures. To measure the influence of the taxonomic model, the raw 7-mer oligonucleotide frequencies were used as an additional profile space. 

For the evaluation of the metagenome universe, all archaeal, bacterial and viral genomes were downloaded from the National Center for Biotechnology Information (NCBI) FTP server [37,38]. They were complemented by 53 Eukaryotic genomes, 33 from diArk [19] and 20 from NCBI. As described in [9], we also included virus-enriched metagenomes to manage the underrepresentation of viral diversity in genome databases. For each reference, the Pfam profile was calculated and profiles with low coverage (<1000 Pfam hits) were excluded from downstream analysis. In addition, we removed similar profiles (correlation of >99% on phylum level) to reduce reference profile redundancy. This process reduced the number of reference genomes to 2199, including 157 Archaea, 1617 Bacteria, 50 Eukaryota, 273 Viruses and 102 viral metagenomes. 

#### 3.2.3. Mixture-of-Pathways

The Mixture-of-Pathways (MoP) model extends the taxonomic mixture model to a statistically adequate modeling of the metabolic potential of metagenomes [20]. MoP is based on a mixture model of pathways for the estimation of relative KEGG pathway abundances. To overcome computationally intense homology searches, we used the MoP-Pro approach introduced in [20]. MoP-Pro implements a shortcut to estimate the metabolic profile of a metagenome by linking the taxonomic profile of the metagenome to a set of pre-computed metabolic reference profiles. Here, organism-specific metabolic profiles in terms of KEGG Ortholog groups are computed for all bacterial and archaeal genomes in the KEGG database. Combining these organism-specific profiles according to the taxonomic profile of the metagenome, we estimate the relative pathway abundances by the posterior probabilities of the metabolic mixture model described in [20]. 

#### 3.2.4. Protein Alignment Using a DNA Aligner (PAUDA) Annotation

The protein alignment using a DNA aligner (PAUDA) approach performs a protein database search [21]. PAUDA converts all protein sequences into pseudo DNA by mapping the amino acid alphabet onto a four-lettered alphabet. Then the read aligner Bowtie2 is used to compare the pseudo DNA reads with a pseudo DNA database. The statistical significance of matches is calculated based on protein alignments of the backtranslated protein sequences. PAUDA runs ∼10,000 times faster than BLASTX, while achieving about one-third of the assignment rate of reads to KEGG orthology groups. In this study, PAUDA was used to perform a search against the functional sequence database including all KEGG Orthologs of bacterial and archaeal origin available in the KEGG database (Release 64.0) and protein domain families in the Pfam database (Release 27). Here we extracted all full length sequences labeled according to their Pfam ID from the ’Pfam-A.full’ multiple alignment file. The homology search was executed in --fast mode with default parameters. In the case of multiple matches, only the best hit is considered. 

### 3.3. Nearest Neighbor Analysis

In our study, we introduce the concept of identifying neighbors of a query metagenome within a database of annotated reference metagenomes based on their taxonomic or functional profiles. For evaluation of the neighbor detection we performed a leave-one-out cross-validation on all metagenome profiles using a *k*-nearest-neighbor search with *k* = 10. As an accuracy measure we counted the fraction of profiles in the neighborhood with the same habitat label as the query profile. Here, we used the habitat assignments of publicly available metagenomes (see above) as obtained from their annotation. We utilized different linear and nonlinear metrics in the profile space to calculate the distances between pairs of metagenomes. 

Let x and y be taxonomic or functional profile vectors of two metagenomes, then the City block (or *L*_1_) distance between x and y can be calculated according to

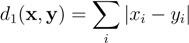
(1)
Note that in case of relative abundances, *i.e.*, $\sum_{i}x_{i}=\sum_{i}y_{i}=1$, the City block distance corresponds to the Bray–Curtis dissimilarity, which is widely used in ecology for comparison of two assemblages [22]. Analogously to the City block metric, the Euclidean (or *L*_2_) distance can be computed according to

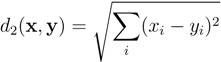
(2)
A standardized version of the Euclidean distance can be obtained by normalizing each profile dimension with respect to its standard deviation, *i.e.*,

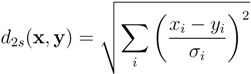
(3)


The Pearson correlation coefficient

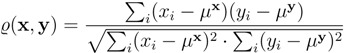
(4)
between two metagenome profiles can be utilized as a distance according to *d_P_*(x, y) = 1 − *ϱ*(x, y). Similarly, Spearman’s rank correlation coefficient defines a distance metric *d_S_*(x, y) = 1 −*ϱ*(x̂, ŷ), whereby x̂ corresponds to a representation of the profile x ith values converted to ranks. 

Finally, we used the Jensen–Shannon divergence, a symmetrized version of the Kullback–Leibler divergence dKL, to measure the distance between metagenomes. The Jensen–Shannon divergence is defined by

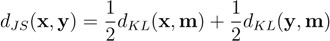
(5)
whereby $d_{KL}(\text{x,y})=\sum_{i}x_{i}ln(\frac{x_{i}}{y_{i}})$ and $\text{m}=\frac{1}{2}(\text{x}+\text{y})$. To prevent numerical problems we excluded profile dimensions that did not contribute any counts from the computation. 

### 3.4. Dimensionality Reduction

For visualization of the high-dimensional metagenome profile data we compared different dimensionality reduction methods: principal component analysis (PCA, [23]), classical multidimensional scaling (MDS, [23]), Sammon mapping [24] and unsupervised kernel regression (UKR, [12]). Sammon mapping and MDS were both based on City block (*L*_1_) distances and UKR was used with the *L*_1_-kernel. The iterative optimization schemes of the Sammon and UKR methods were initialized with the MDS and *L*_1_-kernel PCA, respectively. For computation we used the dimensionality reduction [39] and UKR [40] toolboxes in MATLAB. No additional parameters (hyperparameters) were required by any of the chosen methods. The resulting 2D coordinates of the dimensionality-reduced representation of all metagenomes were used for the neighborhood evaluation based on an Euclidean distance. 

## 4. Conclusions

The focus of our study has been on the comparison of unsupervised methods for metagenome similarity search. The aim was not to introduce a particular method that has been tuned to provide the best classification performance for a given labeling of the data. If the prediction of certain categories is the main objective, then supervised methods can be used that explicitly utilize the label information for parameter optimization [27]. However, our results indicate that the labeling of metagenomic data may also give rise to uncertain categories that are not well represented in terms of profile similarity. Therefore, a supervised approach may be adequate for a rather specific task if well-defined categories and reliable labels are available, for instance to predict a certain disease in a medical context. In contrast, an unsupervised approach to metagenome similarity computation can be more general and may even provide the potential for the discovery of novel or unexpected relationships. Furthermore, the performance of unsupervised methods does not depend on the quality of labels and mislabeled data may even be identified by inconsistent neighborhoods in profile space. On the other hand, metagenomic database retrieval would largely benefit from high-quality metadata and therefore the increasing acceptance of the “Minimum Information about a Metagenome Sequence” (MIMS) specification [28] will multiply the utility of profile-based metagenome comparison. We are aware of the fact that the coarse habitat-oriented labeling that we used in our comparison can only give a first impression of what is actually possible with profile similarity detection. However, the results indicate that a sequence feature-based identification of meaningful metagenomic neighbors is possible and computationally efficient for a wide range of profiles and distance metrics. Although we identified certain combinations that should not be used, in general no single metric or profiling method systematically outperformed the other methods in terms of the neighborhood accuracy. This implies that the profile space and the distance measure can in principle be chosen to allow a convenient interpretation of results in terms of the underlying features. In this context, protein families and metabolic pathways can provide a biologically more powerful representation than oligonucleotide-based features. With biologically meaningful profile features at hand our approach for neighbor identification allows subsequent in-depth analysis such as the identification and interpretation of features which contribute most to the distance between two metagenomes. Therefore, we have started to integrate a *k*-nearest-neighbor search based on protein domain frequency features in the CoMet-Universe server [41], which already implements some of the techniques that we have evaluated in our study. 

## Supplementary Files

Supplementary File 1
