# Supplementary material for: Exploring Neighborhoods in the Metagenome Universe
Source: Int J Mol Sci. 2014 Jul 14;15(7):12364–78. doi: 10.3390/ijms150712364 (PMC4139848; doi:10.3390/ijms150712364)

Supplementary Information

1. Supplementary Methods

The sequence files for the Human Microbiome Project (HMP) website (http://www.hmpdacc.org/HMASM/) contains 764 clinical study-related files, for 750 of which we were able to download, extract and process the corresponding sequence data. Each dataset is composed of two different types of read files, whereby we only selected the paired-end reads which passed the HMP quality control filter. To reduce the computational demands for very large files, we randomly selected 200 MB of data from files exceeding this limit. In addition, sequences with 32 identical starting nucleotides were excluded because they most likely represent homo-polymeric regions or sequencing-related artifacts.

2. Supplementary Tables

Table S1. Confusion matrix of neighborhood evaluation on complete metagenome universe for different habitats according to UProC protein domain profiles and City block metric.Values represent rounded percentages and entries lower than 0.5 are omitted.

**Habitat**	**Aquatic**	**Extreme**	**Food**	**Fossil**	**Host-ass.**	**Terrestrial**	**Virus-enr.**	**Airways**	**Skin**	**UG tract**	**Oral**	**Feces/GI**
Aquatic	93	2	-	-	1	4	1	-	-	-	-	-
Extreme	76	7	-	4	-	11	-	-	-	-	1	-
Food	-	-	75	-	4	-	-	2	2	-	2	16
Fossil	-	-	-	99	-	-	-	-	-	-	-	-
Host-associated	3	-	-	-	36	2	-	1	-	-	3	54
Terrestrial	7	1	-	1	2	77	1	-	-	-	-	11
Virus enriched	33	2	-	1	3	6	49	1	-	-	2	4
Airways	-	-	-	-	-	-	-	89	5	-	6	-
Skin	-	-	-	-	-	-	-	59	39	-	2	-
UG tract	-	-	-	-	-	-	-	-	-	94	5	1
Oral	-	-	-	-	-	-	-	-	-	-	100	-
Feces/GI tract	-	-	-	-	10	1	-	-	-	-	-	89

Table S2. Number of metagenomic datasets for (**A**) Human Microbiome Project and (**B**) comprehensive metagenome collection ("metagenome universe") according to body sites/habitats.

**Body Site**	**# Samples**
Airways	86
Gastrointestinal (GI) tract	134
Oral	344
Skin	26
Urogenital (UG) tract	50
Total	640

(**A**)

**Habitat**	**# Samples**
Aquatic	223
Extreme	14
Food	12
Fossil	285
Host-associated	108
Terrestrial	29
Virus-enriched	45
Airways	92
Skin	16
UG tract	57
Oral	283
Feces + GI tract	581
Total	1745

(**B**)

Table S3. Dimensionality of different profiling methods. Theoretical dimensionality ("all") and actual number of non-zero dimensions based on HMP datasets.

**Method**	**All**	**Non-Zero**
MetaPhlAn	718	711
Taxy-Pro	2199	2045
Taxy-Oligo	2199	1074
7-mer	16384	16384
KO	13328	13324
GO	61	61
UProC	15660	11510
HUMAnN	297	297
MoP-Pro	285	285

3. Supplementary Figures

Figure S1. Neighborhood accuracy on HMP datasets for different profiling methods and metrics.

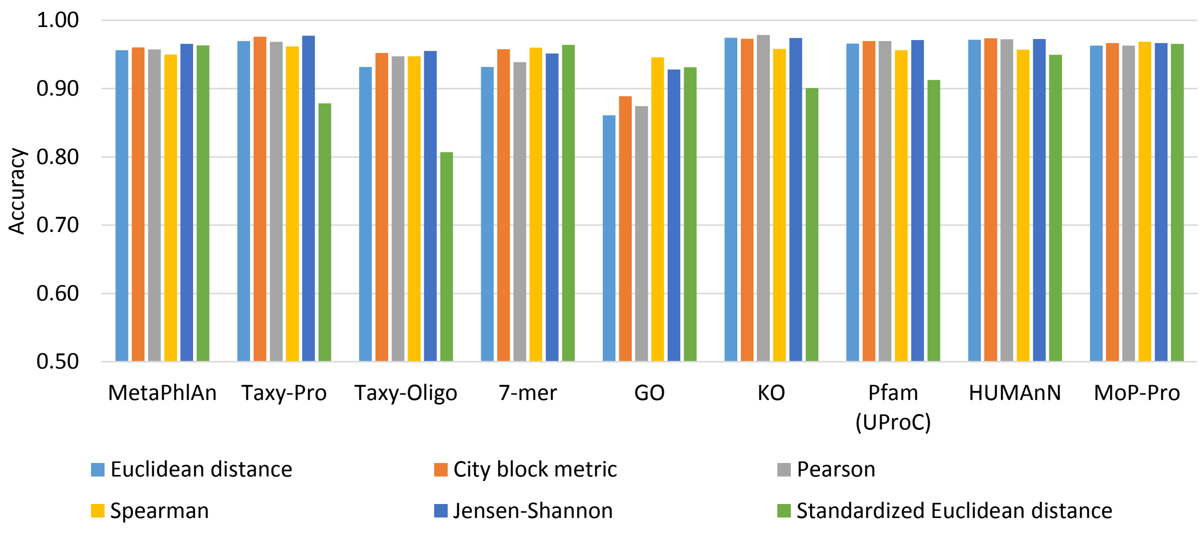

Figure S2. Neighborhood accuracy on HMP body sites for different profiling methods using the City block metric.

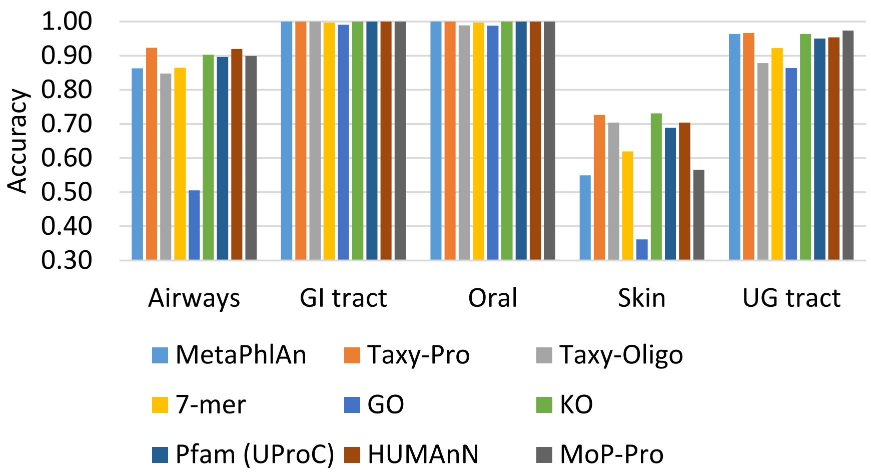

Figure S3. Neighborhood accuracy on HMP body sites for different metrics and several profiling methods. (**A**) Accuracy of distance metrics with average/minimum/maximum over nine different profiling methods; (**B**) Accuracy of profiling methods with average/minimum/maximum over six different metrics.

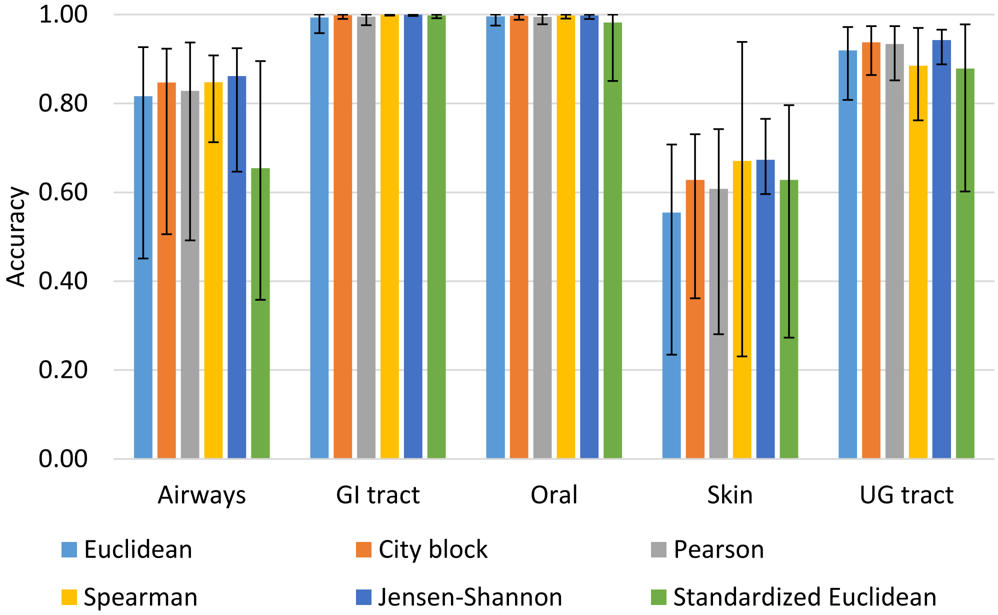

(**A**)

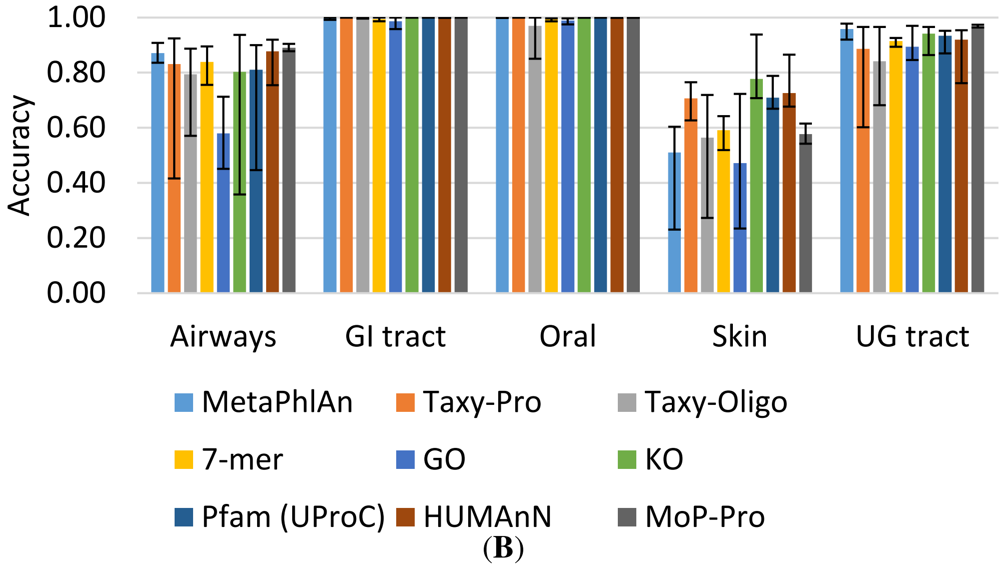

(**B**)

Figure S4. Neighborhood detection accuracy on HMP body sites for different profiling methods using the Spearman metric. (**A**) Body site-specific accuracy averaged over seven different profiling methods; (**B**) Accuracy for different body sites according to nine different profiling methods.

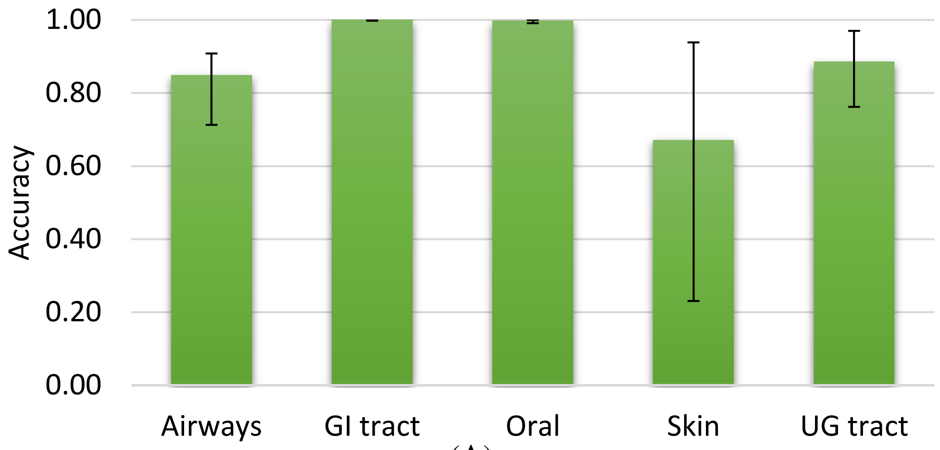

(**A**)

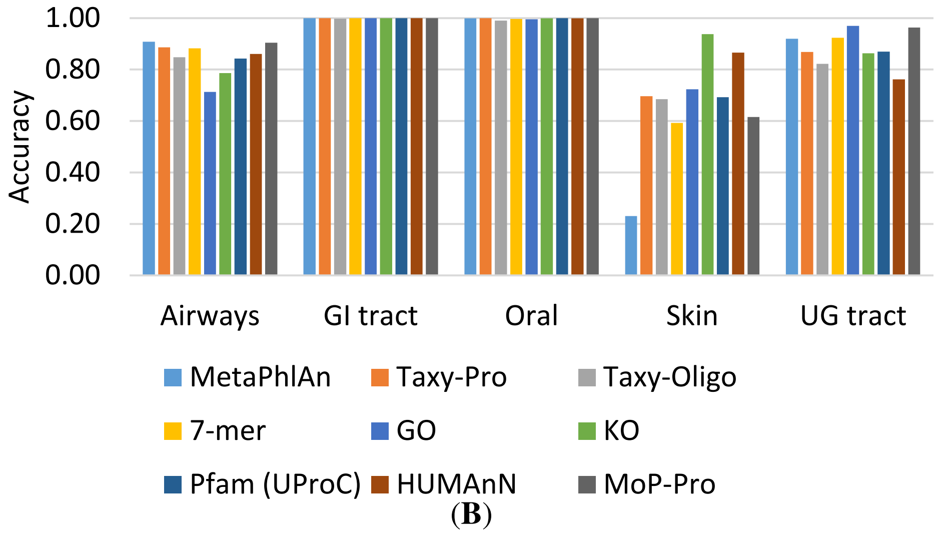

(**B**)

Figure S5. Multi-dimensional scaling (MDS) plot of metagenome neighborhoods and dendrograms from hierarchical clustering analysis (HCA) for two HMP datasets from the "Airways" categories. (**A**,**B**) MDS/HCA for dataset SRS023847 with 6 correct and 4 incorrect neighbors from k-nearest neighbor analysis based on UProC domain profiles and City block metric; (**C**,**D**) MDS/HCA for dataset SRS015051 with 4 correct and 6 incorrect neighbors.

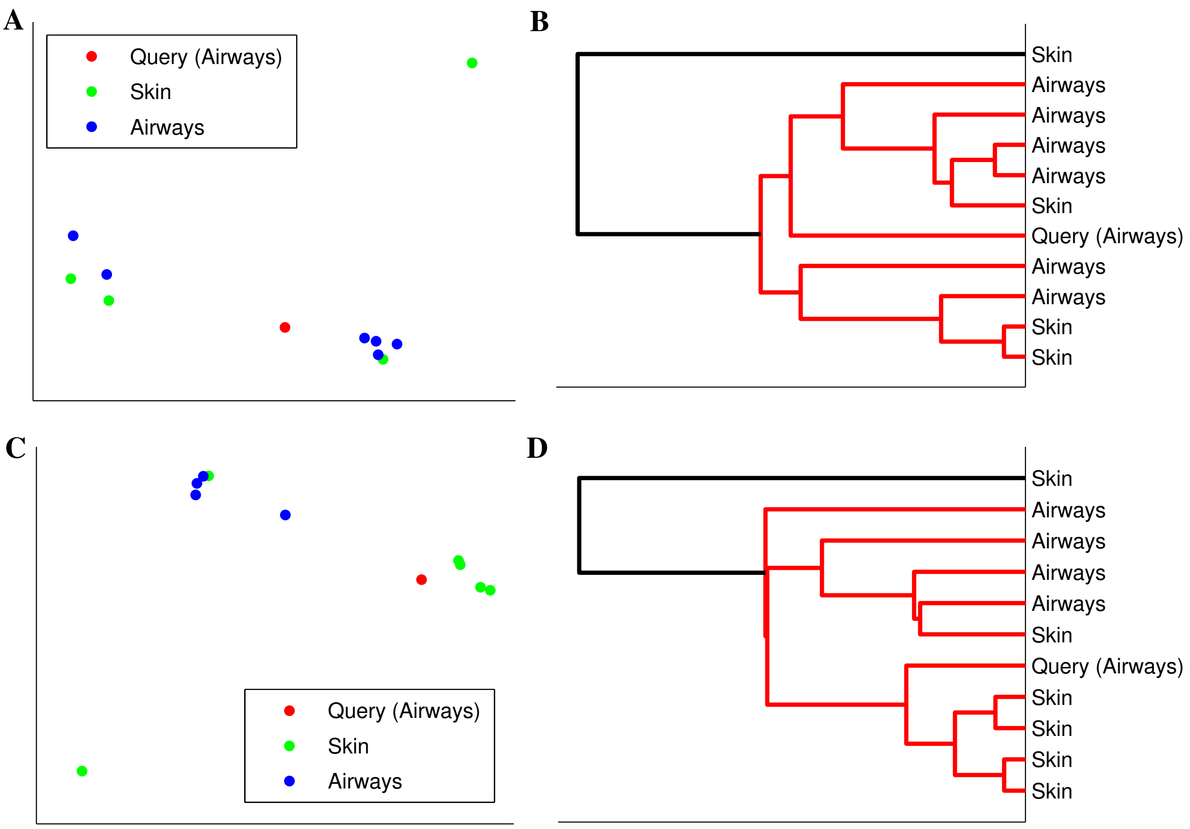

Figure S6. Neighborhood accuracy on metagenome universe collection for different methods and habitats using the City block metric.

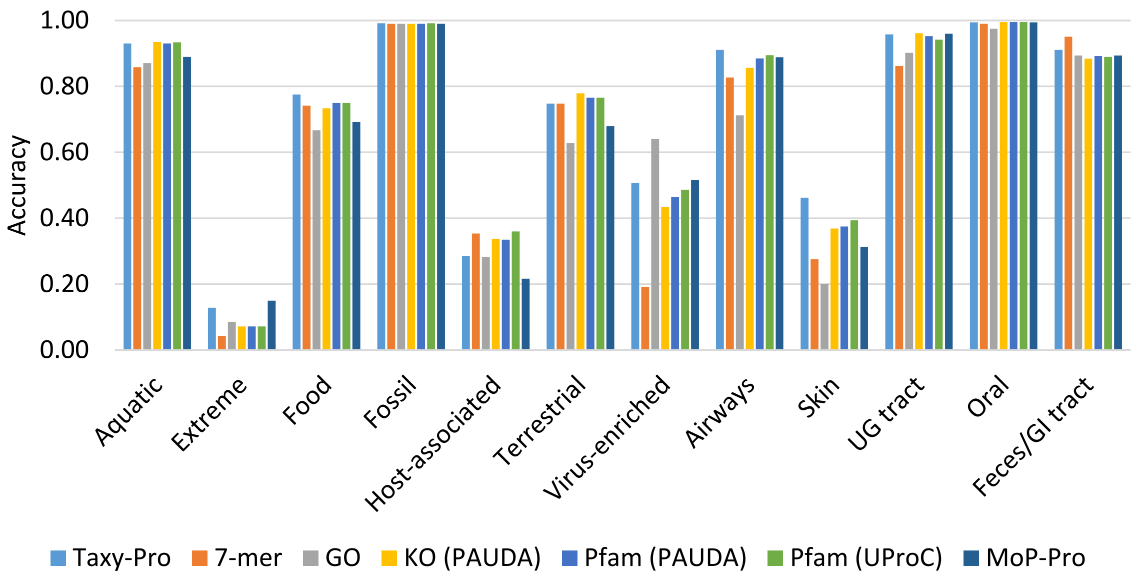

Figure S7. Neighborhood accuracy on metagenome universe collection for different methods and habitats using the Spearman metric. (**A**) Habitat-specific accuracy averaged over seven different profiling methods; (**B**) Accuracy for different habitats according to seven different profiling methods.

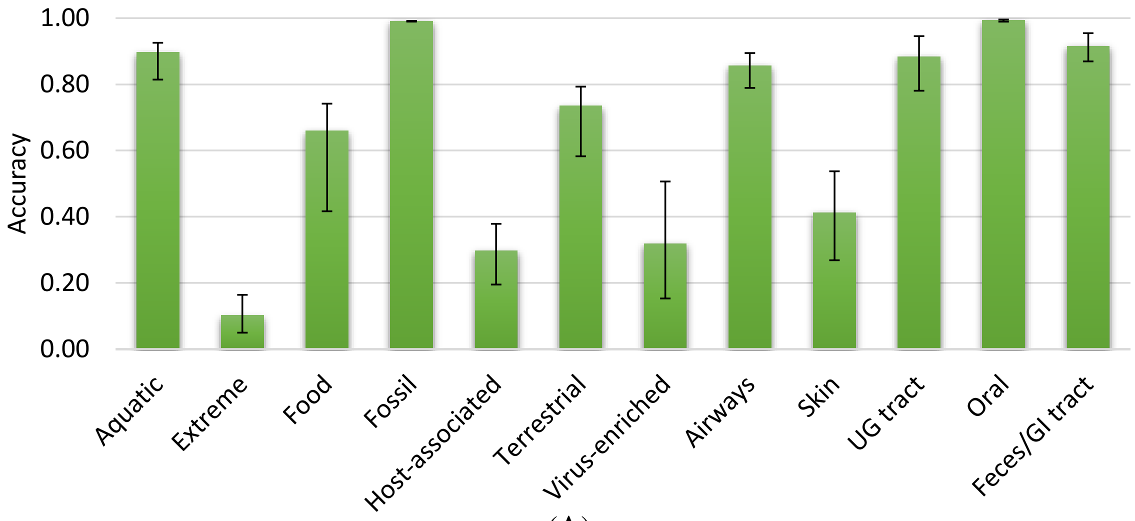

(**A**)

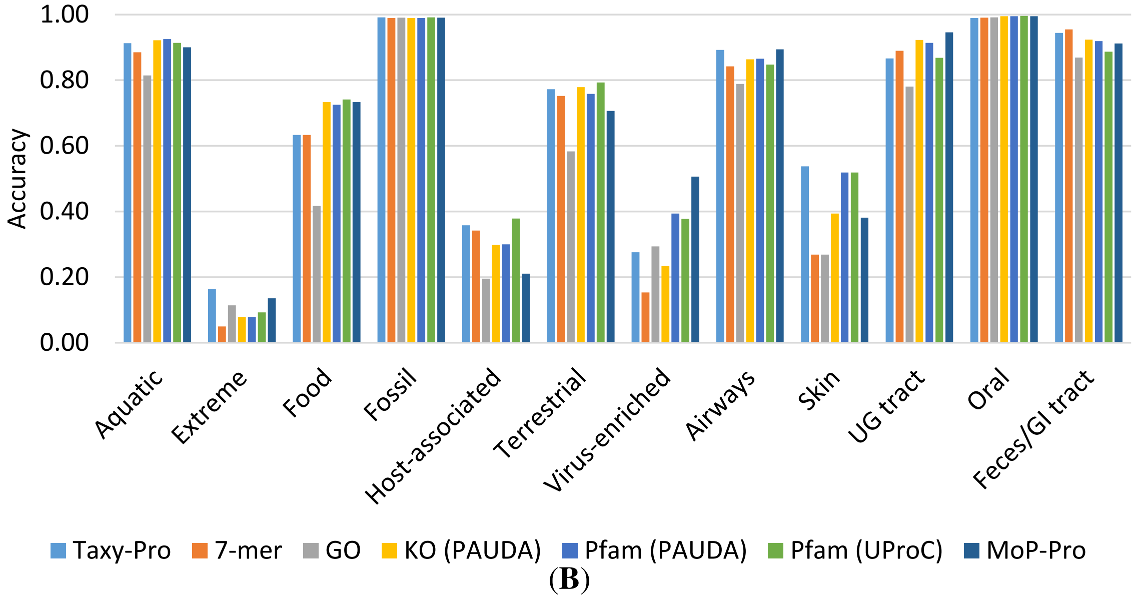

(**B**)

Figure S8. Principal component analysis plot of metagenome universe dataset without viral metagenomes using UProC protein domain profiles. Dimension-specific variance in parantheses.

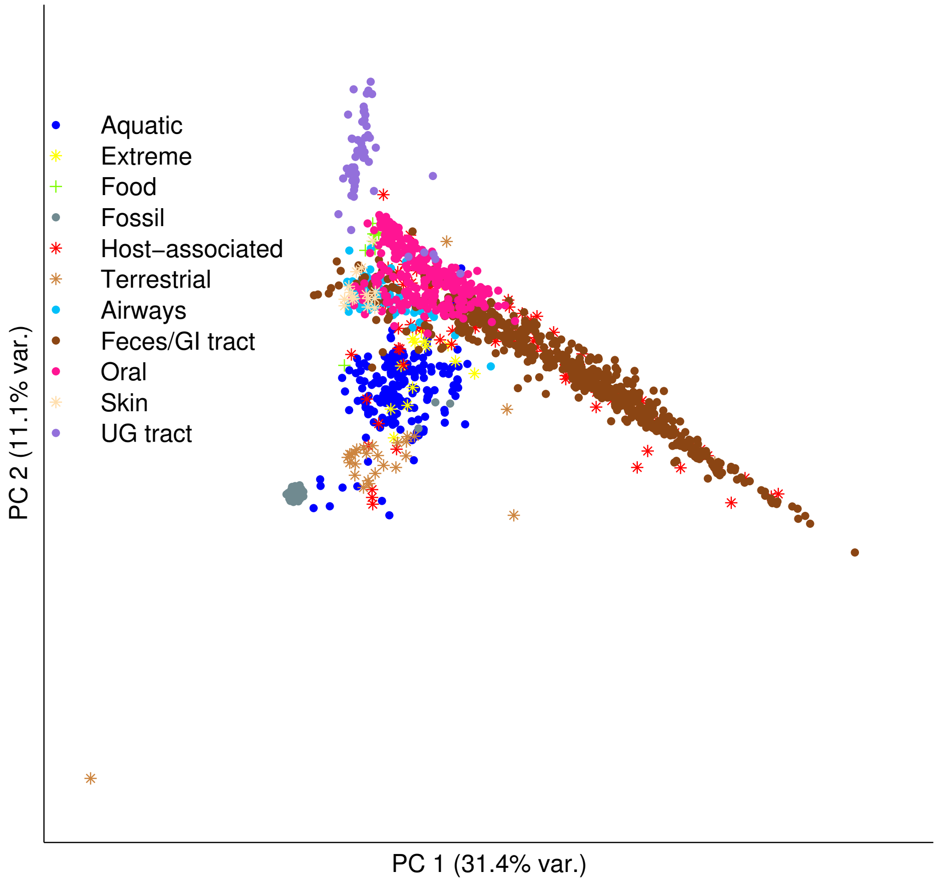

Supplement: Supplementary File 1 [file ijms-15-12364-s001.zip › Supplementary Information.pdf]
